# The acceptability and feasibility of an anxiety reduction intervention for emergency department patients with non-cardiac chest pain

**DOI:** 10.1080/13548506.2016.1144891

**Published:** 2016-02-29

**Authors:** Rosie Webster, Andrew Robert Thompson, Paul Norman, Steve Goodacre

**Affiliations:** ^a^ Research Department of Primary Care and Population Health, University College London, Royal Free Campus, Rowland Hill Street, London, NW3 2PF, UK; ^b^ Department of Psychology, University of Sheffield, UK; ^c^ Department of Psychology, University of Sheffield, Western Bank, Sheffield, S10 2TN, UK; ^d^ Department of Psychology, University of Sheffield, UK; ^e^ School of Health and Related Research, University of Sheffield, UK

**Keywords:** Non-cardiac chest pain, anxiety, CBT, self-help, acceptability

## Abstract

Despite good physical prognosis, patients who receive a diagnosis of non-cardiac chest pain (NCCP) may experience persistent pain and distress. While cognitive-behavioural interventions have been found to be effective for this group, they are difficult to deliver in busy emergency department (ED) settings. Addressing the acceptability and relevance of self-help interventions is an important initial step in addressing this need. This study sought to examine the acceptability and relevance of an evidence-based self-help intervention for ED patients with persistent NCCP and anxiety. Patient (interviews: *N* = 11) and specialist chest pain nurse (focus group: *N* = 4) views on acceptability and feasibility were examined. Data were analysed using thematic analysis. Patients and nurses reported that there was a need for the intervention, as stress and anxiety are common among patients with NCCP, and provision of psychosocial support is currently lacking. Both patients and nurses reported that the intervention was relevant, acceptable, and potentially useful. Some changes to the intervention were suggested. Nurses reported that the intervention could be used within the existing staff resources available in an ED setting. This study represents an important first step towards developing a brief self-help intervention for ED patients with NCCP and anxiety. Further research should seek to determine the efficacy of the intervention in a pilot trial.

## Introduction

Acute chest pain accounts for approximately 700,000 emergency department (ED) attendances each year in England and Wales (Goodacre et al., 2005), but between 30 and 60% of these patients do not receive a cardiac diagnosis (Eken et al., 2010; Mayou & Thompson, 2002). Despite having excellent long-term prognosis (Papanicolaou et al., 1986), many patients with non-cardiac chest pain (NCCP) experience increased anxiety, reduced quality of life, further chest pain, and high service use (e.g. Goodacre, Mason, Arnold, & Angelini, 2001; Hadlandsmyth, Rosenbaum, Craft, Gervino, & White, 2013; Smeijers et al., 2013; Webster, Norman, Goodacre, & Thompson, 2012). NCCP also has indirect economic costs through lost work days (Eslick, Coulshed, & Talley, 2002). Current guidelines for the treatment of NCCP recommend the provision of reassurance that test results are negative (National Institute for Health and Care Excellence, 2010). However, this may not be enough to address patients’ concerns (McDonald, Daly, Jelinek, Panetta, & Gutman, 1996), and may be ineffective due to continuing anxiety (Donkin et al., 2006). As a result, it may be necessary to provide psychological interventions (Kisely, Campbell, Yelland, & Paydar, 2012).

Previous psychological interventions, based on cognitive behavioural therapy (CBT), have been shown to be efficacious in reducing pain and psychological distress in patients with NCCP (Beek et al., 2013; Jonsbu, Martinsen, Morken, Moum, & Dammen, 2013; Kisely et al., 2012). However, CBT can be difficult to implement due to limited time and expertise in ED settings (Esler & Bock, 2004; Kisely et al., 2012). As a result, a stepped approach to treatment has been proposed (Kisely et al., 2012; Mayou, Bass, & Bryant, 1999) with self-help forming an initial step (Hirai & Clum, 2006). Such materials have been effective for anxiety disorders (Lewis, Pearce, & Bisson, 2012) and may be particularly useful within this setting (Esler et al., 2003). Brief information leaflets for chest pain patients within the ED have been shown to have an effect on psychological outcomes but no impact on chest pain (Arnold, Goodacre, Bath, & Price, 2009). The need for brief interventions that impact on both psychological and physical outcomes is apparent.

The design and content of the present intervention were based on findings from previous research based on the Common Sense Model of Illness Representations (Leventhal, Meyer, & Nerenz, 1980). Webster, Norman, Goodacre, Thompson, and McEachan (2014) found that the belief that one’s NCCP was due to psychological causes (e.g. stress) was associated with increased anxiety and depression which, in turn, were related to more frequent chest pain at follow-up. In addition, interviews with NCCP patients who were experiencing continued anxiety and chest pain highlighted a lack of understanding of their chest pain (Webster, Thompson, & Norman, 2015). These findings indicate that, contrary to previous suggestions (Esler & Bock, 2004), NCCP patients may be accepting of psychological explanations, and therefore open to psychological interventions.

The aim of this study was to assess the acceptability of a self-help intervention for patients experiencing persistent NCCP and high levels of anxiety. The views of NCCP patients and specialist chest pain nurses were sought.

## Methods

### Design

The study comprised two parts: a focus group with specialist chest pain nurses and individual interviews with patients eligible to receive the intervention. Ethical approval was obtained from an NHS Research Ethics Committee.

### Participants

All specialist chest pain nurses working within an ED in Northern England (*N* = 7) were invited to participate, of whom four participated (100% female; aged 29–50). As the focus group was conducted during working hours, it was not possible for all nurses to participate. A convenience sample of patients with NCCP and elevated anxiety was recruited from the same ED. The inclusion criteria were: (i) diagnosed NCCP, (ii) no other significant health/mental health complaint, (iii) aged 18 or over, (iv) no known CHD, (v) able to comprehend English, (vi) score above the threshold for mild anxiety on the GAD-7 (see below). Participants were invited to participate once ED staff confirmed that there was no cardiac cause for their pain. After being given information regarding the study, interested participants were asked to complete the GAD-7. Those who scored ≥ 5 on the GAD-7 were invited to complete a consent form. Participants were recruited to the study over a 10-week period between February and April 2012, between the hours of 09.30 and 17.30, Monday to Friday. During this period, 40 patients were identified as suitable by the specialist chest pain nurses and subsequently approached regarding participation. Of these patients, 9 declined participation (reasons unknown), 4 took an information sheet but did not contact the researcher, 4 were later found to have a cardiac cause for their pain, and 9 scored below 5 on the GAD-7. Of the 14 patients recruited into the study, 3 subsequently contacted the researcher expressing their wish to withdraw from the study (prior to interview). The final sample comprised 11 participants.

#### Data collection

The data were collected by the first author. The focus group and interviews were recorded and transcribed for analysis. Participants in the interviews and the focus groups were informed that the leaflet was designed to be provided to patients upon receiving a diagnosis of NCCP.

Focus group: The nurses were given the intervention materials at the start of the focus group, and a semi-structured interview schedule was used to assess understanding, relevance, acceptability, and potential for use (see Table 1). The focus group lasted 75 min and was conducted by the first author, who was known to the nurses from a previous study, but did not work in the ED.

**Table 1.** Focus group questions (Nurses).

Topic	Questions
General opinion	What is your general opinion of the leaflet?
Understanding	Is the leaflet understandable to you?
	What do you think about the length of the material?
	Do you think there is anything missing from the material?
	Is there anything within the leaflet that you didn’t like (if so will be followed up by why)?
Acceptability	How suitable do you think the intervention would be for chest pain patients?
	How appropriate is it in targeting the important issues for this patient group?
	How would you feel about delivering the intervention leaflet yourselves?
Promise	How effective do you think it would be in treating patients with anxiety-related chest pain?
Feasibility	What are the costs of delivering an intervention like this?
	How are the information leaflets for patients currently in place used?
	At the moment it is all in paper format. What do you think about this? Might it be delivered in other ways?

Interviews: At the start of the interview, participants were given the intervention materials and asked to comment on them as they viewed them, using a ‘think aloud’ methodology (Boren & Ramey, 2000; Ericsson & Simon, 1984) as used in other studies developing interventions (e.g. Sadasivam et al., 2011; Yardley, Morrison, Andreou, Joseph, & Little, 2010). Participants were then asked about the acceptability and usability using a semi-structured interview schedule (see Table 2). Interviews lasted 30–90 min.

**Table 2.** Interview questions (Patients).

Topic	Questions
General opinion	What is your general opinion of the leaflet?
Understanding	Is the material written in a way that’s clear and understandable?
	Do you think the length of the material is OK?
	Do you think there is anything missing from the information and suggested techniques?
	Is there anything within the leaflet that you didn’t like?
Acceptability	Do you think the leaflet is relevant to you, as a non-cardiac chest pain patient?
	Do you think it could meet your needs?
	Do you think it could help you to reduce or cope with your chest pain?
Promise	Would it be something you’d like to try?
	Would you consider using these techniques on a regular basis, in order to help cope with your chest pain?
	After reading the material, do you feel capable of using/doing the techniques described in it?
	Would you show it to anybody else (in your family)?
	Do you think the format of it – a leaflet – is useful?

#### Materials

GAD-7: This 7-item measure for assessing Generalised Anxiety Disorder (the GAD-7) has high internal consistency, good test-retest reliability, and good validity (Spitzer, Kroenke, Williams, & Lowe, 2006). A cut-off score of ≥ 5 indicates mild anxiety.

The Intervention: The intervention was developed by the first and second authors, and was assessed for comprehensibility by a Patient and Public Involvement Emergency Care Forum. The Flesch (1948) reading ease score for the intervention was 75.3 (classed as ‘fairly easy’).

The intervention content was developed using the findings of previous research (Webster et al., 2014, 2015), which demonstrated that psychological morbidity is strongly related to outcomes, and that NCCP patients often lack a clear understanding of the causal mechanisms of chest pain. The content of previous successful face-to-face CBT interventions for NCCP (Esler et al., 2003; Jonsbu, Dammen, Morken, Moum, & Martinsen, 2011; Potts, Lewin, Fox, & Johnstone, 1999; van Peski-Oosterbaan et al., 1999) was also used to guide development. The intervention built on a prior information only leaflet (Arnold et al., 2009), with the inclusion of techniques to target anxiety. The intervention booklet comprised three sections:

Section one explained potential causes of NCCP (adapted from Arnold et al., 2009). Stress was introduced as both a causal and maintaining factor of pain. The relationships between feelings, thoughts, behaviours, and physical sensations were explained using a diagram that users could complete (adapted from Padesky & Mooney, 1990). A pain diary for recording associated thoughts and feelings was provided. Section two included techniques to cope with pain, and manage stress (Mindful breathing, guided imagery, progressive muscle relaxation, and physical activity). Section three provided guidance on planning to use the techniques, using implementation intentions (Gollwitzer, 1999), whereby users make a specific ‘if-then’ plan for using the techniques.

### Data analysis

All interviews were transcribed by an independent transcriber, and checked for accuracy by the first author. Data were analysed using thematic analysis (Braun & Clarke, 2006; Joffe, 2012). Data from the nurses and the patients were initially analysed separately and then compared in order to identify similarities/differences. A record was kept of all stages of data collation and analysis. The second author audited the analysis process by reviewing the content of each analytic stage (Mays & Pope, 2000; Spencer & Ritchie, 2012).

## Results

### Participants

Four specialist chest pain nurses participated in the focus group (100% female; aged 29–50). The most effective focus groups contain between 6 and 8 participants; however, this can be reduced where the participants have expertise (Krueger, 1995), as in the present case. Eleven NCCP patients met the inclusion criteria and agreed to participate (see Table 3). This number of participants is sufficient for think aloud methodology (Protheroe, Blakeman, Bower, Chew-Graham, & Kennedy, 2010; Yardley, Miller, Teasdale, Little, & Primit, 2011).

**Table 3.** Characteristics of participants (*N* = 11).

Variable	Median	Range
Age	46	30–70
Age leaving education	16	15–40
GAD-7 score	8	5–17
*Variable*		*n*
Gender	Male	5
	Female	6
Highest qualification achieved	None	1
	School/College	6
	University	4
Interview location	Participant’s home	8
	ED	2
	University	1

### Themes

Four broad themes were identified: perceived benefits of the intervention, content of the intervention, implementation, and suggested changes.

#### Perceived benefits of the intervention

Both the nurses (*N*) and NCCP patients (*P*) were positive about the intervention, and described it as having the potential to be useful, helpful, understandable, informative, beneficial, and simple to use.

All participants described a need for the intervention due to the widespread problem of anxiety and lack of existing resources. Nurses described the intervention as helpful in clarifying causes of chest pain, and patients described the intervention as having a normalising effect.

N01: It’s really good for us that we can provide this information to patients.

P09: It tells you things in here that your Doctor wouldn’t even tell you … you know how it says relax yourself … you don’t get that from your Doctor, you just get tablets.

P03: the whole document here, it has inferred that people do suffer from chest pains and it’s not necessarily cardiac-related, it’s stress, I think it probably would be sort of helpful to let people know [that].

#### Content of the intervention

The majority of participants felt that the content of the intervention was relevant and that the multiple elements of the intervention meant it could be relevant to a variety of people.

P08: Yeah definitely with me it’s stress, I would think, without question.

N03: Some people may well not want to interact with it and write in it and whatever, but will find the [...] techniques really useful.

Participants felt that section one sufficiently explained the causes of chest pain. Both groups rated the pain diary positively, although some patients did not like the idea of having to write.

P06: Yeah that’s a good idea, keeping a diary. That’s something you say you’ll do if somebody suggested it, but you wouldn’t necessarily [do it], but actually for somewhere to write it down makes sense.

Most participants disliked the hot-cross bun diagram, as they found it either difficult to understand or irrelevant.

P06: The only one thing that I keep going back to is [the diagram], I don’t know why, I didn’t understand [it].

N01: I don’t like the little diagram. I just think it’s confusing, sorry. We have them in nursing all the time and I hate them. I just don’t feel that that really adds anything.

Participants were positive about the relaxation techniques. The nurses thought it was especially good that users were offered a choice of techniques. The patients found the section encouraging physical activity particularly valuable. Nurses noted patients often worry whether exercise was safe.

N01: It’s good [that] you do give different options, because some people won’t be able to do some but will be able to try the others.

P06: You do it as you’re reading […] I was doing it then […] yeah it is quite relaxing to just take everything in. Mmm interesting, [it’s] not something I’ve thought about..

Most patients spoke positively about potentially using the implementation intention plans to remember to use the techniques, although some indicated that they did not like the language used.

P11: I do need a plan, I do need to make more of a routine for the areas I can and this needs to be in a routine.

P02: Yeah the plan it switches me off totally […] it’s not for me that type of thing.

#### Implementation

Some felt that extra guidance and support might be needed, although there was a concern among the nurses regarding a lack of time for this. It was suggested that a short script might be beneficial. The nurses stated that the intervention was feasible to deliver.

P05: I think that people … to do that they will need the encouragement of going back, the feedback of more helpful suggestions or whatever from [a health professional]. A lot will start to [think] ‘I don’t see the point in that, I’m not going back there, they won’t know if I’ve done it.

N02: Most patients we get will understand it, and you’ll know the ones we get through who won’t and you can just take 5 min out and just briefly [explain it to them].

Some nurses were concerned that ED staff might inadvertently give the intervention to someone with cardiac problems, suggesting that training would be a necessary part of implementation. Patients were also concerned that the intervention could cause people to delay seeking care; however, the nurses reported that the provision of appropriate discharge advice would avoid this risk.

N04: Even if we educate the nurses to give these [out], the doctors still should give […] the standard advice.

P09: That’s why you have to do something about it because if you just sit here and think ‘oh I’ve got a right chest pain, it’s only stress that’s causing it’ and it’s something else, then you either get through [the] day and get it sorted or […] you’re dead aren’t you when it’s a [heart problem].

#### Suggested changes

Some nurses felt that the wording in section three (implementation intentions) was patronising.

N03: ‘Some people might find this kind of thing helpful’, as opposed to saying, ‘after you’ve tried these relaxation techniques, select one or two and make a plan in the boxes below’. I don’t know, I just feel like it’s a little bit patronising.

Patients suggested a number of potential additions to the intervention, such as case studies, advice on how to fit the techniques into everyday life, and information about physiological stress-pain mechanisms.

P01: It would be nice if there was some information and scenarios from other people, you know little paragraphs of other people experiencing the same, so that you don’t feel alone.

P03: You don’t really say why people have chest pains because of stress … if you could put that in a light hearted way, I mean not [use] long words of Latin, just explain [it].

Some patients described using alternative methods of relaxation and the inclusion of guidance to increase the frequency of use of such existing methods could therefore be included.

P07: When I’m stressed […] I just take Paracetamol or whatever and go and have a lie down, have a sleep, just chill out, you know.

Participants were asked about the possibility of including audio and online components in the intervention and all were positive about the inclusion of audio support, with some patients mentioning this spontaneously, indicating that it could increase accessibility, and be useful to guide relaxation practice.

N04: When you’re trying to do the relaxation things but you’re trying to read it through at the same time, when you’re trying to close your eyes and do things, it’d be nice if somebody [said] ‘now do this, now do that’ without you having to read it.

P10: It would help people perhaps who have got difficulty to reading […] personally, years and years ago I had a relaxation [cassette tape], and it was a man reading it with the most relaxing voice I have ever heard and it did, it made you relax just listening to him talking.

Some participants stated that making the intervention available online would be useful. However, there was also a concern that this might exclude people.

N02: Not everybody has a computer at home, so you’d probably be excluding quite a lot of patients, and it’s probably the ones who are socially deprived who would probably get a lot of benefit from it.

P06: Yeah definitely I mean I pointed out when I read [the suggested links] that I tend to go on the internet […] so I probably would..

## Discussion

This study sought to assess the acceptability of a self-help intervention for patients with NCCP and anxiety. The findings from both the focus group with specialist chest pain nurses and the interviews with patients with NCCP and anxiety revealed positive views of the potential utility of the intervention.

The patients were positive about at least part, if not all, of the intervention content. Interestingly, nearly all participants were negative about the CBT diagram (Padesky & Mooney, 1990). This appeared mainly due to a lack of understanding and suggests that, if included, further information about the CBT model might be required (Padesky & Mooney, 1990). However, the evidence for the efficacy of this diagram in improving outcomes is weak (Bieling & Kuyken, 2003), and so it could be removed from the intervention. Furthermore, given this diagram’s frequent use (Whittington & Grey, 2014), its acceptability within self-help warrants wider investigation.

Considering changes to the intervention, the incorporation of narrative accounts from patients was suggested. This would emphasise how patients are not alone in their suffering and could also help to increase the empathic tone of the intervention (Richardson & Richards, 2006). It was also suggested that the intervention should include more detailed physiological explanations of causal mechanisms, which have been found to improve reassurance in medically unexplained symptoms (Dowrick, Ring, Humphris, & Salmon, 2004).

Both nurses and patients positively endorsed the inclusion of an audio element. Combining media in self-help can significantly improve efficacy (Gould & Clum, 1993; Lewis et al., 2012). The inclusion of an online element was also seen as positive; however, there were some concerns about accessibility and cost. A significant proportion of the UK population have Internet access (Office for National Statistics, 2013), so concerns regarding accessibility may not be substantiated. Furthermore, an online version would allow for easy updating of material, inclusion of media files, tailored content, and wider dissemination.

Given the acceptability of the present intervention, and the acknowledgement of stress and anxiety as a cause of the pain in the current sample, the present intervention has the potential to enhance outcomes in patents with NCCP. Nonetheless, the nurses and patients generated a range of ideas for improving the intervention. As such, the present study highlights the importance of incorporating user feedback, from both patients and health care professionals, in the development phase of an intervention. The use of Think Aloud methods, combined with focus groups and interviews, provides a strong methodological approach for obtaining user feedback. A number of the suggested changes will be made (i.e. removing the CBT diagram, adding accounts of patient’s experiences, including an audio component) in order to improve the intervention and make it more useful and acceptable to users.

There are some study limitations that should be noted. First, whilst the patient sample was purposively selected on the basis of having persistent NCCP and elevated levels of anxiety, it is unlikely to be fully representative of the patient population. Second, the first author was involved in both the development of the intervention and conducting the interviews, which may have resulted in some bias. However, the interview was guided by a semi-structured schedule, and participants were encouraged to provide constructive criticism. Third, views were only obtained from specialist nursing staff working in the ED, and not other staff (e.g. doctors). In the study setting, patients with chest pain were almost entirely managed by the specialist chest pain nurses. As such, the nurses were best placed to comment on the suitability of the intervention, given their extensive expertise and experience in working with chest pain patients, and the fact that they would be likely to deliver the intervention. However, other EDs will have different procedures for managing chest pain. This may therefore limit the transferability of the findings.

In summary, this study suggests that a CBT-based self-help intervention is acceptable, feasible, and understandable for patients with NCCP and elevated anxiety, and for the nurses who care for them. The intervention shows promise and has the scope to be implemented in a stepped care model for patients with persistent NCCP with associated anxiety. Further research is needed to test the efficacy of the modified intervention.

## Disclosure statement

The authors declare that they have no conflict of interests.

## Funding

The first author conducted this research as part of her PhD, which was funded by the Economic and Social Research Council and the Medical Research Council.

## Supplementary materials

Supplemental data for this article can be accessed at http://dx.doi.org/10.1080/13548506.2016.1144891

## Supplementary Material

Supplemenatry_Material.docx

## References

[CIT0001] Arnold J., Goodacre S., Bath P., Price J. (2009). Information sheets for patients with acute chest pain: Randomised controlled trial. *British Medical Journal*.

[CIT0002] Beek M., Oude Voshaar R., Beek A., Zijderveld G. V., Visser S., Speckens A., Balkom A. (2013). A brief cognitive-behavioral intervention for treating depression and panic disorder in patients with noncardiac chest pain: A 24-week randomized controlled trial. *Depression and Anxiety*.

[CIT0003] Bieling P. J., Kuyken W. (2003). Is cognitive case formulation science or science fiction?. *Clinical Psychology: Science and Practice*.

[CIT0004] Boren M. T., Ramey J. (2000). Thinking aloud: Reconciling theory and practice. *IEEE Transactions on Professional Communication*.

[CIT0005] Braun V., Clarke V. (2006). Using thematic analysis in psychology. *Qualitative Research in Psychology*.

[CIT0006] Donkin L., Ellis C. J., Powell R., Broadbent E., Gamble G., Petrie K. J. (2006). Illness perceptions predict reassurance following a negative exercise stress testing result. *Psychology & Health*.

[CIT0007] Dowrick C. F., Ring A., Humphris G. M., Salmon P. (2004). Normalisation of unexplained symptoms by general practitioners: a functional typology. *British Journal of General Practice*.

[CIT0008] Eken C., Oktay C., Bacanli A., Gulen B., Koparan C., Ugras S. S., Cete Y. (2010). Anxiety and depressive disorders in patients presenting with chest pain to the emergency department: A comparison between cardiac and non-cardiac origin. *Journal of Emergency Medicine*.

[CIT0009] Ericsson K., Simon H. (1984). *Protocol analysis: Verbal reports as data*.

[CIT0010] Esler J. L., Barlow D. H., Woolard R. H., Nicholson R. A., Nash J. M., Erogul M. H. (2003). A brief cognitive-behavioral intervention for patients with noncardiac chest pain. *Behavior Therapy*.

[CIT0011] Esler J. L., Bock B. C. (2004). Psychological treatments for noncardiac chest pain – Recommendations for a new approach. *Journal of Psychosomatic Research*.

[CIT0012] Eslick G. D., Coulshed D. S., Talley N. J. (2002). Review article: The burden of illness of non-cardiac chest pain. *Alimentary Pharmacology & Therapeutics*.

[CIT0013] Flesch R. (1948). A new readability yardstick. *The Journal of Applied Psychology*.

[CIT0014] Gollwitzer P. M. (1999). Implementation intentions – Strong effects of simple plans. *American Psychologist*.

[CIT0015] Goodacre S., Cross E., Arnold J., Angelini K., Capewell S., Nicholl J. (2005). The health care burden of acute chest pain. *Heart*.

[CIT0016] Goodacre S., Mason S., Arnold J., Angelini K. (2001). Psychologic morbidity and health-related quality of life of patients assessed in a chest pain observation unit. *Annals of Emergency Medicine*.

[CIT0017] Gould R. A., Clum G. A. (1993). A meta-analysis of self-help treatment approaches. *Clinical Psychology Review*.

[CIT0018] Hadlandsmyth K., Rosenbaum D. L., Craft J. M., Gervino E. V., White K. S. (2013). Health care utilisation in patients with non-cardiac chest pain: A longitudinal analysis of chest pain, anxiety and interoceptive fear. *Psychology & Health*.

[CIT0019] Hirai M., Clum G. A. (2006). A meta-analytic study of self-help interventions for anxiety problems. *Behavior Therapy*.

[CIT0020] Joffe H., Thompson D. H. A. (2012). Thematic analysis. *Qualitative research methods in mental health and psychotherapy: A guide for students and practitioners*.

[CIT0021] Jonsbu E., Dammen T., Morken G., Moum T., Martinsen E. W. (2011). Short-term cognitive behavioural therapy for non-cardiac chest pain and benign palpitations: A randomised controlled trial. *Journal of Psychosomatic Research*.

[CIT0022] Jonsbu E., Martinsen E. W., Morken G., Moum T., Dammen T. (2013). Change and impact of illness perceptions among patients with non-cardiac chest pain or benign palpitations following three sessions of CBT. *Behavioural and Cognitive Psychotherapy*.

[CIT0023] Kisely, Campbell L. A., Yelland M. J., Paydar A. (2012). Psychological interventions for symptomatic management of non-specific chest pain in patients with normal coronary anatomy. *Cochrane Database of Systematic Reviews*.

[CIT0024] Krueger R. A. (1995). The future of focus groups. *Qualitative Health Research*.

[CIT0025] Leventhal H., Meyer D., Nerenz D., Rachman S. (1980). The common sense model of illness danger. *Medical psychology*.

[CIT0026] Lewis C., Pearce J., Bisson J. I. (2012). Efficacy, cost-effectiveness and acceptability of self-help interventions for anxiety disorders: Systematic review. *The British Journal of Psychiatry*.

[CIT0027] Mayou R., Bass C. M., Bryant B. M. (1999). Management of non-cardiac chest pain: From research to clinical practice. *Heart*.

[CIT0028] Mayou R., Thompson D. (2002). Treatment needs of patients admitted for acute chest pain. *Journal of Psychosomatic Research*.

[CIT0029] Mays N., Pope C. (2000). Qualitative research in health care – Assessing quality in qualitative research. *British Medical Journal*.

[CIT0030] McDonald I. G., Daly J., Jelinek V. M., Panetta F., Gutman J. M. (1996). Opening Pandora's box: The unpredictability of reassurance by a normal test result. *British Medical Journal*.

[CIT0031] National Institute for Health and Care Excellence (2010). Chest pain of recent onset: Assessment and diagnosis of recent onset chest pain or discomfort of suspected cardiac origin. http://www.nice.org.uk/nicemedia/live/12947/47938/47938.pdf.

[CIT0032] Office for National Statistics (2013). Internet access – household and individuals.

[CIT0033] Padesky C. A., Mooney K. A. (1990). Clinical tip: Presenting the cognitive model to clients. *International Cognitive Therapy Newsletter*.

[CIT0034] Papanicolaou M. N., Califf R. M., Hlatky M. A., McKinnis R. A., Harrell F. E., Mark D. B., Pryor D. B. (1986). Prognostic implications of angiographically normal and insignifcantly narrowed coronary-arteries. *American Journal of Cardiology*.

[CIT0035] Potts S. G., Lewin R., Fox K. A. A., Johnstone E. C. (1999). Group psychological treatment for chest pain with normal coronary arteries. *Qjm-Monthly Journal of the Association of Physicians*.

[CIT0036] Protheroe J., Blakeman T., Bower P., Chew-Graham C., Kennedy A. (2010). An intervention to promote patient participation and self-management in long term conditions: Development and feasibility testing. *BMC Health Services Research*.

[CIT0037] Richardson R., Richards D. A. (2006). Self-help: Towards the next generation. *Behavioural and Cognitive Psychotherapy*.

[CIT0038] Robinson N. (1999). The use of focus group methodology – with selected examples from sexual health research. *Journal of Advanced Nursing*.

[CIT0039] Sadasivam R. S., Delaughter K., Crenshaw K., Sobko H. J., Williams J. H., Coley H. L., Houston T. K. (2011). Development of an interactive, web-delivered system to increase provider – Patient engagement in smoking cessation. *Journal of Medical Internet Research*.

[CIT0040] Smeijers L., van de Pas H., Nyklicek I., Notten P. J., Pedersen S. S., Kop W. J. (2013). The independent association of anxiety with non-cardiac chest pain. *Psychology & Health*.

[CIT0041] Spencer L., Ritchie J., Harper D., Thompson A. R. (2012). In pursuit of quality. *Qualitative research methods in mental health and psychotherapy: A guide for students and practitioners*.

[CIT0042] Spitzer R. L., Kroenke K., Williams J. B. W., Lowe B. (2006). A brief measure for assessing generalized anxiety disorder – The GAD-7. *Archives of Internal Medicine*.

[CIT0043] van Peski-Oosterbaan A. S., Spinhoven P., van Rood Y., van der Does J. W., Bruschke A. V., Rooijmans H. G. (1999). Cognitive-behavioral therapy for noncardiac chest pain: A randomized trial. *American Journal of Medicine*.

[CIT0044] Webster, Norman P., Goodacre S., Thompson A. (2012). The prevalence and correlates of psychological outcomes in patients with acute non-cardiac chest pain: A systematic review. *Emergency Medicine Journal*.

[CIT0045] Webster R., Norman P., Goodacre S., Thompson A. R., McEachan R. R. C. (2014). Illness representations, psychological distress and non-cardiac chest pain in patients attending an emergency department.. *Psychology & Health*.

[CIT0046] Webster R., Thompson A. R., Norman P. (2015). Everything’s fine, so why does it happen? A qualitative investigation of patients’ perceptions of non-cardiac chest pain. *Journal of Clinical Nursing, Online first*.

[CIT0047] Whittington A., Grey N. (2014). *How to become a more effective CBT therapist: Mastering metacompetence in clinical practice*.

[CIT0048] Yardley L., Miller S., Teasdale E., Little P., Primit T. (2011). Using mixed methods to design a web-based behavioural intervention to reduce transmission of colds and flu. *Journal of Health Psychology*.

[CIT0049] Yardley L., Morrison L. G., Andreou P., Joseph J., Little P. (2010). Understanding reactions to an internet-delivered health-care intervention: Accommodating user preferences for information provision. *BMC Medical Informatics and Decision Making*.

