# Supplementary material for: The acceptability and feasibility of an anxiety reduction intervention for emergency department patients with non-cardiac chest pain
Source: Psychol Health Med. 2016 Feb 29;22(1):1–11. doi: 10.1080/13548506.2016.1144891 (PMC5105082; doi:10.1080/13548506.2016.1144891)
Supplement: Supplemenatry_Material.docx [file cphm_a_1144891_sm1877.docx]

| **Coping with chest pain** |
| --- |
| Information and guidance to help you understand and cope with your chest pain |
|  |
| Developed by:  Ms. Rosie Webster, Psychology PhD Researcher  Dr. Andrew Thompson, Clinical and Health Psychologist  Dr. Paul Norman, Reader in Health Psychology  Prof. Steve Goodacre, Professor of Emergency Medicine  University of Sheffield, 01.12.2011 |
|  |
|  |
|  |
|  |

The tests that have been done today have shown that we cannot identify any cardiac (heart-related) cause for the chest pain that you have been experiencing. This is not uncommon. Many people who come into the emergency department with chest pain are diagnosed with so-called ‘non-cardiac chest pain’, that is, chest pain where no heart-related cause has been found.

**What is non-cardiac chest pain?**

Some people experience chest pain, either as a one-off or regularly, for which no serious cardiac cause can be found. This does not mean that the pain doesn’t exist or is not distressing. The pain is real and can be quite upsetting and scary.

**Why have I been given this leaflet?**

Even though people with non-cardiac chest pain have been told that nothing has been found to be wrong with their heart, some still experience chest pain and continue to be worried about it. In this leaflet we hope to reassure you and give you ways to cope with any further pain you experience. It has three sections:

1. *‘Recognising what causes non-cardiac chest pain’:* Here we explain some reasons why you might be experiencing pain, and talk about how stress can trigger the pain or make it worse.
2. ‘*Ways to deal with chest pain’:* Here we give you proven methods to cope with pain and reduce the effect of stress. This includes some techniques to help you to relax.
3. ‘*Making a plan to use your new coping methods’*: Here, we will show you how to make a plan to use the techniques described in section 2.

**Section one: Recognising what causes my non-cardiac chest pain**

*‘Now I can relate it to the stress levels whereas before I thought about it but I didn’t really relate it’*

Your doctor may have already explained that you have had non-cardiac chest pain and that this can be associated with stress. So how might this work?

The quotes in the speech bubbles are things non-cardiac chest pain patients have said. They talk about the relationships that they have noticed between stress, anxiety, and pain.

Several things can trigger the symptoms of non-cardiac chest pain, including:

- Tension and strain in the **muscles** in the chest

*‘Yeah when I’m a bit anxious about things and…I’ve noticed a bit when I’m a bit wound up’*

- **Breathing** incorrectly, including breathing too fast or straining the chest muscles while breathing
- Spasm, tightening, or inflammation of the **oesophagus** (food pipe)

*‘I had chest pain the other evening but I had had quite a stressful day at work’*

- **Stress can play a role in causing tension in the muscles in the chest, and breathing incorrectly. This chest pain can then also cause more stress, or make the stress worse.**

Many of us experience stress in our daily life. We often do not realise how this stress can have a physical impact on us. A lot of the causes listed above, such as muscle tension, can be triggered by stress. Often, we might not even realise that we are tense until we experience symptoms like chest pain.

**The vicious cycle of pain, worry, tension, pain**


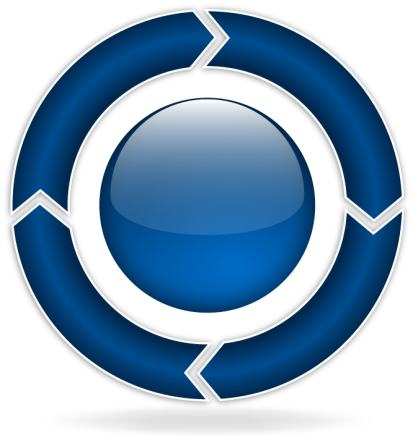
Chest pain can be worrying. This worry may trigger bodily tension, which can cause further pain. This can then set up a ‘vicious cycle’, of pain, worry, and tension - as shown in figure 1 on the next page.

Figure 1 (on the following page) shows how thoughts, behaviours, physical sensations, and feelings can all be linked. Pain might cause feelings like stress and anxiety, lead you to have worrying thoughts, and cause you to avoid activities, but these can all make the pain worse.

Some of the examples in the diagram might be familiar, but do put your own examples in the blank spaces in the boxes. Write about your own thoughts and feelings when you get your pain. Write about what physical sensations you experience. Also, add in details of any ways in which you have changed your behaviour because of your pain (e.g. avoiding certain activities like strenuous exercise). Doing this is important as it will help you see the connections involved between your thoughts, feelings, behaviour and experience of non cardiac chest pain. This is the first step towards breaking the vicious cycle described above.

**Avoiding activity may feed the vicious cycle**

Avoiding activity, like exercise, can be linked to feelings, tension and worry as shown in Figure 1. Chest pain might make you want to avoid activities because you worry it might make the chest pain worse. However, avoiding physical activity can make things worse. Exercise is actually a very good way to release stress and tension and is proven to improve health.

**Thoughts**

- ‘Am I having a heart attack?’

- ‘What if this pain doesn’t ever go away?’

- _________________

- _________________

**Physical sensations**

**-** Pain

- Breathlessness

- Chest tightness

- _________________

- _________________

**Feelings**

- Stress

- Anxiety

- Fatigue

- _________________

- _________________

**Behaviours**

- Avoiding physical activity

- Avoiding everyday activities

- _________________

- _________________

Figure 1. Adapted from Padesky and Mooney (1990)

**Making the connections for yourself**

You may find it helpful to make a diary, where every time you experience pain, write down how you are thinking and feeling at the time. This might help you to make some connections between how you’re feeling and the pain you are experiencing. Use the diary below to record when you get pain, what you were doing at the time, and any thoughts you had in your head at the time.

| **When did the pain come on?** | **What were you doing when the pain came on?** | **How were you feeling when the pain came on? What were you thinking?** |
| --- | --- | --- |
| *E.g., Tuesday, 2pm, just after lunch which I had eaten at my desk.* | *E.g., Working on my computer.* | *E.g., I was tense because I was thinking that I had too much to get done and I’d miss my deadlines.* |
|  |  |  |
|  |  |  |
|  |  |  |
|  |  |  |
|  |  |  |
|  |  |  |
|  |  |  |
|  |  |  |
|  |  |  |
|  |  |  |

**Section two: Ways to deal with chest pain**

We hope section one has showed you how there are many other causes for chest pain, and how stress might both cause, and be brought on by, chest pain. Now we want to share with you some tried and tested techniques to help you to cope with chest pain and to reduce it. These include things that you can do when the pain comes on, and things that you can do daily that may help to reduce your stress levels, which can help reduce your chest pain.

**Increasing physical activity**

As it says above, exercise can be a good way to release stress and improve health. So being more active is also likely to actually help reduce chest pain. Make a list of activities that you can fit into your daily life. These may be activities that you have been avoiding because of your pain. You can include everyday activities like household chores on the list. You should also try to include some activities you enjoy, like walking the dog, playing games or sport. Evidence suggests that just half an hour of exercise a day can make a big difference to physical and emotional health. Exercise does not have to involve running and jumping, low intensity activity like the examples below are also very good. Make your own list of personal activities below:

| *E.g., Walking the dog* |
| --- |
| *E.g., Using the stairs at work* |
|  |
|  |
|  |
|  |
|  |

Now think about when you could fit these activities into your life over the next week. Fit them into the diary below, to try and make sure you are active at least once a day. It might help if you show your diary to someone close to you, for example a partner or a close friend, who might encourage you to stick to your plans, or better still might agree to join in with you!

| **Day** | **Activity** | | |
| --- | --- | --- | --- |
|  | **What?** | **Where?** | **When?** |
| *Example* | *Walking the dog* | *In the park* | *2pm, after lunch* |
| Monday |  |  |  |
| Tuesday |  |  |  |
| Wednesday |  |  |  |
| Thursday |  |  |  |
| Friday |  |  |  |
| Saturday |  |  |  |
| Sunday |  |  |  |

**Techniques for reducing bodily tension and increasing relaxation**

We have included here a number of proven techniques that can be used to reduce bodily tension. Read through each one and then have a go at trying them. For them to be helpful they will need to be practiced regularly as just like any new skill they will take a while to learn. After trying them all, you might want to practise just one or two of them as described in section 3.

**Mindful breathing:** This is a way of focussing on your breathing, on purpose, in the present moment. It is a way of clearing your mind and focussing on the present and is akin to meditation that has been used by many cultures over time to achieve a state of well-being. With practice you will find that it helps you to relax and be less concerned about pain. You can then use it when the pain comes on, and as part of your daily routine to reduce stress. To start with try setting aside a specific amount of time, around 20 minutes, to practice mindful breathing.

- The goal of mindful breathing is to help develop a peaceful state of mind, allowing thoughts and feelings to simply come and go without getting caught up in them. The idea is to be able to observe yourself. However, it’s not simple to do – our minds naturally wander! So it is important to practice and to not criticise yourself when your mind does wander. Self-critical thoughts can be particularly hard to observe and let pass through your mind without engaging with them.

Try to follow the steps below:


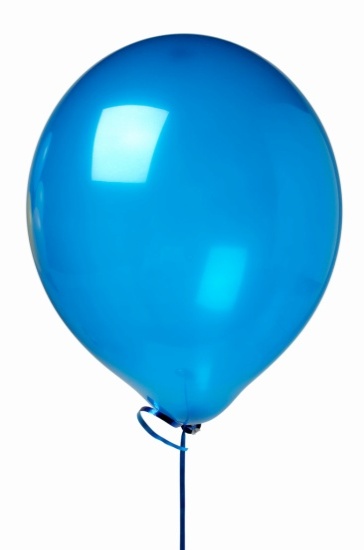


- - Sit comfortably, with your eyes closed .
  - Start to notice your own breathing.
  - Notice the sensations in your stomach as it rises and falls with each breath.
  - Thoughts, feelings, and external noises will come into your mind. Simply notice these things, almost as you would passing traffic, trying not to think any more about them. Allow yourself to simply notice them and then, gently bringing your attention back to your breathing.
  - You may feel sleepy when you do this, that’s OK, but again simply notice this feeling and bring your attention back to your breathing.

**Guided imagery:** Guided imagery is different from mindful breathing in so far as the focus here is on creating a vivid relaxing scene in your minds eye. This can help you to feel both more relaxed and more positive, and as such it can be another good technique for managing pain.

Again it is important to practice this technique before you could expect to use it when you are in pain. Try to practice for about 20 minutes.

Try to follow the steps below:

- Get yourself into a comfortable and quiet place where you ideally won't be disturbed. Take a couple of minutes to focus on your breathing.
- Imagine a place where you can feel calm, peaceful and safe. It may be a place you've been to before (perhaps a holiday scene or family event), or somewhere you go to actually relax (perhaps a favourite chair), or even a fantasy place (perhaps a beautiful meadow or beach).
- Focus on creating the scene in your mind’s eye.
- Notice all of the details.
- Notice the colours, who’s with you, the sounds, smells, even the physical sensations.
- Notice the sounds that are around you, perhaps there is the quiet noise of the waves or wind etc.
- Picture the smells - perhaps there is the smell of the sea etc.
- Imagine the physical sensations - perhaps you can imagine the warmth of the sun on your face, the feeling of the breeze on your skin etc..
- If the image fades (and it will), don’t worry or get frustrated, simply refocus on the details, to imagine almost what it would be like to be there.
- Now whilst you're in this place, choose a name that captures the feeling and say this to yourself under your breath. The word might be something like relaxed, chilled, calm, peaceful, safe, or some other such word.
- Eventually using this word alone will bring on the feelings, but this will take practice!

**Muscle relaxation:** When we are tense regularly our muscles can hold this tension without us even being aware of this. This sort of tension can eventually show itself as pain. Setting time aside to identify muscle tension (by tensing areas for a few seconds) and then relaxing the muscles is an important skill that may help in managing stress and non-cardiac stress pain:

Try the following steps.

- Sit or lie in a comfortable position and try to ensure that you will not be disturbed. As with the other techniques, try to focus solely on the exercise. If other things (thoughts or external noises) enter your mind - try to simply notice them without thinking any more about them.
- Focus now on your head - notice how your forehead feels. Let any tension go, imagining your forehead becoming smooth. Let any tension go from around your eyes, your mouth, your cheeks and your jaw. Let your teeth part slightly and feel the tension go. Now notice the difference in the feeling between tension and relaxation.
- Now focus on your neck and shoulders - tense them and then let them relax. Again notice the difference in the feeling between tension and relaxation.
- Moving to your arms and your hands. Make each arm rigid and make a fist. Now let them go limp. Again notice the difference in the feeling between tension and relaxation.
- Moving your attention to your back. Tense your back and notice how it feels. Then let the tension go and feel yourself sinking down into the chair.
- Now your legs and feet straighten each leg in turn and curl up your feet. Again hold this tension for a few seconds, before letting it go, noticing the difference between tension and relaxation.
- Now scan through your body and if you notice anywhere is tense – repeat the exercise for that area.
- Finally spend a few minutes enjoying the feeling of relaxation, just concentrating on your breathing.

**Section three: Making a plan to use your new coping methods**

It can be very easy to forget to use relaxation techniques, especially when we are feeling stressed or are in pain. It can help if we make a specific plan for when and how we will use these techniques.

For example, to make a plan to use a technique when you experience symptoms, you might write this:

When I

*experience pain*,

then I will

*do mindful breathing*

Now, make a plan to use one of the techniques when you experience symptoms, such as pain, chest heaviness, or stress:

When I experience

____________________________________

then I will

________________________________________________________________

As we said earlier, it is important that you practice these techniques so you feel comfortable doing them. After you have tried all three relaxation techniques, select one or two that you find most helpful. Now make a plan in the box below for when you will practise these techniques. For example:

When I

*go to bed in an evening*

then I will

*practise the muscle relaxation activity*

Now, complete the box below for yourself:

When I ____________________________________

then I will

________________________________________________________________

Now say out loud what you have written in the two boxes a few times until you can remember them without referring back to the sheet. Keep the plans in mind, and use them! Feel free to make more than one plan.

**Further support**

If you would like to learn other ways in which you can cope with stress, anxiety and pain, various self-help resources can be found at the following website:

[www.getselfhelp.co.uk](http://www.getselfhelp.co.uk)

The following organisations can offer information and support for people suffering with high levels of anxiety:

Anxiety UK, 08444 775 774

[www.anxietyuk.org.uk](http://www.anxietyuk.org.uk)

Mind, 0845 766 0163

[www.mind.org.uk](http://www.mind.org.uk)

SANE, 0845 767 8000

[www.sane.org.uk](http://www.sane.org.uk)

If you continue to experience high levels of stress and anxiety, please consult your GP who may be able to refer you to further sources of help, including face-to-face therapy.

If you continue to experience chest pain on a regular basis, and the techniques in this leaflet do not help, please consult your GP.
